# Cerebro Spinal Nerves

**Published:** 1837

**Authors:** 


### IX.—Cerebro-Spinal Nerves.

*Plates of the Cerebro-Spinal Nerves; With references, for the use of Medical Students.* By Paul B. Goddard, M. D., Prosector of Anatomy in the University of Pennsylvania; Member of the Academy of Natural Sciences, of the Philadelphia Medical Society, etc. Philadelphia: J. G. Auner, 1837.

We are indebted to Mr. Auner, the enterprizing publisher, for a copy of the above work, which has just left his prolific press. It comprises twelve well executed lithographs, representing the origin, course, and destination of the nerves which are detached from the cerebro-spinal axis. Most of the plates have been taken from the works of Bell, Swan, and Cloquet, modified in such a manner as to adapt them more fully to the wants of the American student. The references are remarkably concise, and therefore well calculated to prevent confusion; a circumstance which is so apt to happen when they are copious. The author has not encumbered his book with any description of the objects which he has so faithfully represented; and in this respect we conceive he has done wisely, as the student can readily obtain what information he may want upon these points by consulting the admirable treatises of Horner, Cloquet, and Meckel. With either of these works before him, every nerve may be traced upon the plates from its origin at the brain or spinal cord, to its final termination in the various organs and tissues of the body, with as much precision almost as upon the dead subject. By this omission, while the main object of the work is fully attained, another important advantage has been secured, that, namely, of cheapness.

Knowing the great assistance which well executed plates are calculated to afford the student, whilst engaged in unraveling the complex structure of the human fabric, we can confidently recommend Dr. Goddard's work as a safe and convenient companion for the dissecting-room. If plates are useful in any branch of anatomical pursuit, they must be doubly so in the study of the nerves. These textures have always been a stumbling block to the young inquirer, and few, it may be safely asserted, ever obtain a sufficient knowledge of them. We hope the author will continue his labors, and ere long give us a series of plates of the diseases of the nerves.

